# Monitoring response to anti-angiogenic mTOR inhibitor therapy in vivo using ^111^In-bevacizumab

**DOI:** 10.1186/s13550-017-0297-9

**Published:** 2017-05-30

**Authors:** Neel Patel, Sarah Able, Danny Allen, Emmanouil Fokas, Bart Cornelissen, Fergus V. Gleeson, Adrian L. Harris, Katherine A. Vallis

**Affiliations:** 1 Department of Radiology, Churchill Hospital, Headington, OX3 7LE Oxford, UK; 2 CRUK/MRC Oxford Institute for Radiation Oncology, Department of Oncology, University of Oxford, Oxford, UK; 3 Department of Oncology, University of Oxford, Oxford, UK

**Keywords:** Radionuclide, Angiogenesis, Cancer, Bevacizumab, Rapamycin

## Abstract

**Background:**

The ability to image vascular endothelial growth factor (VEGF) could enable prospective, non-invasive monitoring of patients receiving anti-angiogenic therapy. This study investigates the specificity and pharmacokinetics of ^111^In-bevacizumab binding to VEGF and its use for assessing response to anti-angiogenic therapy with rapamycin.

Specificity of ^111^In-bevacizumab binding to VEGF was tested in vitro with unmodified radiolabelled bevacizumab in competitive inhibition assays. Uptake of ^111^In-bevacizumab in BALB/c nude mice bearing tumours with different amounts of VEGF expression was compared to that of isotype-matched control antibody (^111^In-IgG1κ) with an excess of unlabelled bevacizumab. Intratumoural VEGF was evaluated using ELISA and Western blot analysis. The effect of anti-angiogenesis therapy was tested by measuring tumour uptake of ^111^In-bevacizumab in comparison to ^111^In-IgG1κ following administration of rapamycin to mice bearing FaDu xenografts. Uptake was measured using gamma counting of ex vivo tumours and effect on vasculature by using anti-CD31 microscopy.

**Results:**

Specific uptake of ^111^In-bevacizumab in VEGF-expressing tumours was observed. Rapamycin led to tumour growth delay associated with increased relative vessel size (8.5 to 10.3, *P* = 0.045) and decreased mean relative vessel density (0.27 to 0.22, *P* = 0.0015). Rapamycin treatment increased tumour uptake of ^111^In-bevacizumab (68%) but not ^111^In-IgGκ and corresponded with increased intratumoural VEGF_165_.

**Conclusions:**

^111^In-bevacizumab accumulates specifically in VEGF-expressing tumours, and changes after rapamycin therapy reflect changes in VEGF expression. Antagonism of mTOR may increase VEGF in vivo, and this new finding provides the basis to consider combination studies blocking both pathways and a way to monitor effects.

**Electronic supplementary material:**

The online version of this article (doi:10.1186/s13550-017-0297-9) contains supplementary material, which is available to authorized users.

## Background

Angiogenesis is central to tumour growth and invasion [1, 2]. As with all cancer therapies, anti-angiogenic therapy is only effective in a subgroup of patients and hence there is a need to develop new methods to predict and monitor response to treatment. In contrast to histological studies, radionuclide imaging has the advantage of offering non-invasive, prospective longitudinal assessment of angiogenesis, of the whole tumour and metastases, by targeting pathways involved in the process [3]. This is also important because of their toxicity and expense.

The vascular endothelial growth factor (VEGF) pathway is one of the key effectors of angiogenesis and primarily mediated by the interaction of VEGF with the receptor VEGF receptor 2 (VEGFR2) [4, 5]. High VEGF concentration in the blood or in tumour tissue has been associated with an elevated risk of recurrence, metastasis and poor survival [6, 7]. Imaging VEGF provides a tool for non-invasively assessing the levels of VEGF within tumour deposits. One method of imaging of VEGF involves radiolabelling bevacizumab (Avastin), a humanised [8] monoclonal immunoglobulin (IgG1κ) that binds all isoforms of human VEGF [9, 10]. Bevacizumab has been previously labelled with ^89^Zr [11, 12], ^64^Cu [13], ^86^Y [14], ^124^I [15], ^125^I [16] and ^111^In [12] for imaging. Of these, ^111^In- and ^89^Zr-labelled bevacizumab have been tested in clinical trials and have shown decreased tracer uptake in patients treated with anti-angiogenic agents [17, 18].

The inhibitors of the mammalian target of rapamycin (mTOR) have potent anti-angiogenic effect [19]. However, the effect of mTOR inhibitors on tumour vasculature in patients with renal and breast cancer remains unexplored. Also, despite several previous studies, the optimal time of imaging after administration of bevacizumab is unclear. In this context, we report here the results of in vitro and in vivo investigation to test the utility of ^111^In-bevacizumab in the detection of the response to the anti-angiogenic mTOR inhibitor rapamycin, analogues of which (everolimus) are used to treat renal and breast cancer.

## Methods

### Conjugation and ^111^In labelling of bevacizumab

Bevacizumab (25 mg/mL, Roche, USA) buffered with sodium bicarbonate (0.1 M, pH 8.2, Sigma-Aldrich) was reacted with 7-fold molar excess of 2-(4-isothiocyanatobenzyl) diethylenetriamine pentaacetic acid (DTPA, Macrocyclics, Dallas, USA) dissolved in anhydrous dimethyl sulfoxide (DMSO, Sigma-Aldrich) for 45 min at room temperature. Unreacted DTPA was removed by gel-filtration chromatography using a G-50 Sephadex (Sigma-Aldrich) column. Purified DTPA-bevacizumab was buffer-exchanged into sodium citrate (0.1 M, pH 5.0, Sigma-Aldrich) and incubated with ^111^In chloride (1–2 MBq/μg) (PerkinElmer) for 1 h at room temperature. Radiolabelling yield was measured using instant thin layer chromatography (ITLC) in sodium citrate (0.1 M, pH 5.0) and was always >95%.

### Cell lines and xenografts

MDA-MB-231 (human breast adenocarcinoma, triple receptor negative) cell lines were a gift from Dr. Helen Sheldon (Weatherall Institute of Molecular Imaging, University of Oxford, UK). The cell lines were selected from clones infected with a retrovirus to express VEGF: 2F11 (244.7 ± 10.0 pg/mg protein, as determined by ELISA) and IE3 (3.6 ± 0.3 pg/mg protein). LS174T (human colorectal adenocarcinoma), 786-O (human renal adenocarcinoma), and FaDu (laryngeal carcinoma) cell lines were obtained from the American Type Culture Collection (ATCC). All cell lines were cultured in Dulbecco’s modified Eagle’s medium (DMEM) (Sigma-Aldrich) supplemented with 10% foetal bovine serum (FBS) (Gibco), L-glutamine (20 mM), penicillin G (100 u/mL) and streptomycin (100 mg/mL) (Sigma-Aldrich) 37 °C in 5% CO_2_ humidified atmosphere.

### In vitro evaluation

The immunoreactivity of ^111^In-bevacizumab was assessed by competitive binding to VEGF expressed on 2F11 cells. Unlabelled bevacizumab (0.01 to 1000 nM) and 1 nM of ^111^In-bevacizumab were added to wells seeded with cells (2 × 10^5^) and incubated at 4 °C for 2 h. Following removal of supernatant and washing, the cells were lysed using sodium hydroxide (250 μL, 0.1 M) and the radioactivity of the lysate was measured in an automated gamma-counter (Wizard, PerkinElmer).

### In vivo biodistribution, kinetics and specificity

All animal procedures were performed in accordance with the Animals Scientific Procedures Act (ASPA) of 1986 (UK). Female athymic BALB/c *nu/nu* (nude) mice (Charles Rivers) were kept in a pathogen-controlled environment with access to food and water ad libitum. Xenografts were established by subcutaneous injection into the flank of mice with 1 × 10^6^ (FaDu) or 4 × 10^6^ cells (MDA-MB231 IE3, LS 174T and 786-O). Experiments were initiated once tumours reached around 7 mm in diameter. Kinetics of uptake were investigated in LS174T xenograft-bearing mice. The mice were euthanised 1, 3, 5 or 7 days after administration of ^111^In-bevacizumab (5 MBq/3 μg), and the organs and tumours were harvested, weighed and radioactivity measured.

Specificity of uptake was determined by administering either ^111^In-bevacizumab (5 MBq, 3 μg); an isotype-matched, ^111^In-labelled, non-specific antibody, ^111^In-IgG1κ (Southern Biotech) conjugated and labelled using the same method as for ^111^In-bevacizumab (5 MBq, 3 μg); or ^111^In-bevacizumab (5 MBq, 3 μg) plus a hundred fold (300 μg) excess of unlabelled bevacizumab. The animals were euthanised on day 5 when organs and tumour were excised to determine uptake of tracer and for protein and histological analyses.

### The effect of rapamycin on ^111^In-bevacizumab uptake

An initial dose-escalation study was performed to determine the optimal dose of rapamycin in FaDu xenograft-bearing BALB/c mice. FaDu xenografts were chosen as they have moderate VEGF production and relatively consistent tumour growth rates. The mice received 1, 5 or 20 mg/kg of rapamycin or vehicle via intraperitoneal injection daily for 10 days. The mice were assessed for systemic side effects of therapy, and tumour sizes were measured daily. Daily 20 mg/kg rapamycin appeared to have greater growth inhibition than the other doses and did not lead to significant systemic effects in the mice.

Subsequently, FaDu xenograft-bearing mice (*n* = 6–7/group) received rapamycin (20 mg/kg; LC laboratories) or vehicle (DMSO, 5% TWEEN-80 (Sigma-Aldrich), 5% polyethylene glycol 300 (PEG300) (Sigma-Aldrich) and 0.9% NaCl (Braun)) by daily intraperitoneal injection for 10 days. On day 5 of rapamycin therapy, the mice received ^111^In-bevacizumab (5 MBq, 3 μg) or ^111^In-IgG1κ (5 MBq, 3 μg) intravenously. After treatment, the mice were euthanised. Tumour size was measured with callipers, and volume was calculated using the formula: *V* = (*a* × *b* ^2^)/2, where *a* and *b* are the largest and the smallest perpendicular diameters, respectively.

### Autoradiography and immunohistochemistry

After resection, 8-μm frozen tumour sections were cut, applied to slides and then imaged in a storage phosphor-imager (Cyclone Plus, PerkinElmer). The distribution of VEGF and bound bevacizumab in frozen-embedded tumour sections were determined by immunohistochemistry using anti-VEGF (ab46154, Abcam) and anti-IgG (709-176-149, Jackson Laboratories) antibodies, respectively. Images were acquired using a confocal immunofluorescence microscope using ×100 magnification (LSM710, Zeiss).

### VEGF quantification by ELISA

Homogenising buffer (ethylenediaminetetraacetic acid (EDTA, 1.5 mM) (Sigma-Aldrich), 4-(2-hydroxyethyl)-1-piperazineethanesulfonic acid (HEPES, 20 mM) (Sigma-Aldrich) and one protease inhibitor tablet (Complete Mini, Roche) per 10 mL, pH 7.5) was added to frozen tumour samples at 0.02 mL/mg. Following homogenisation, the suspension was centrifuged at 4 °C (3000*g* for 10 min). The supernatant was removed and ultracentrifuged at 4 °C (225,000*g* for 40 min). The supernatant was used for protein analysis. Protein concentrations were determined using the Bio-Rad colorimetric DC protein assay. Tumour VEGF concentration was determined using the VEGF Quantikine kit (R&D systems). VEGF levels were normalised for protein concentration.

### VEGF isoform analysis by Western blotting

Homogenised samples from individual tumours were diluted with PBS to give protein concentrations of 0.25–0.4 mg/mL and run on a 4–12% Bis (2-hydroxyethyl) imino-tris (hydroxymethyl) methane-HCl (Bis-Tris) precast gel (Invitrogen). The following standard proteins were used: VEGF_121_ (4644-VS-010, R&D Systems), VEGF_165_ (293-VE-010, R&D Systems) and VEGF_189_ (ab106307, Abcam). Primary antibodies, rabbit anti-VEGF (sc152, Santa Cruz) and rabbit anti-beta actin (ab8227, Abcam), were added to the blot followed by a secondary antibody, goat, anti-rabbit horseradish peroxidase (HRP) conjugate (656120, Invitrogen). Analysis of blots was performed using ImageJ (National Institutes for Health).

### Vessel analysis

**Fig. 1:**
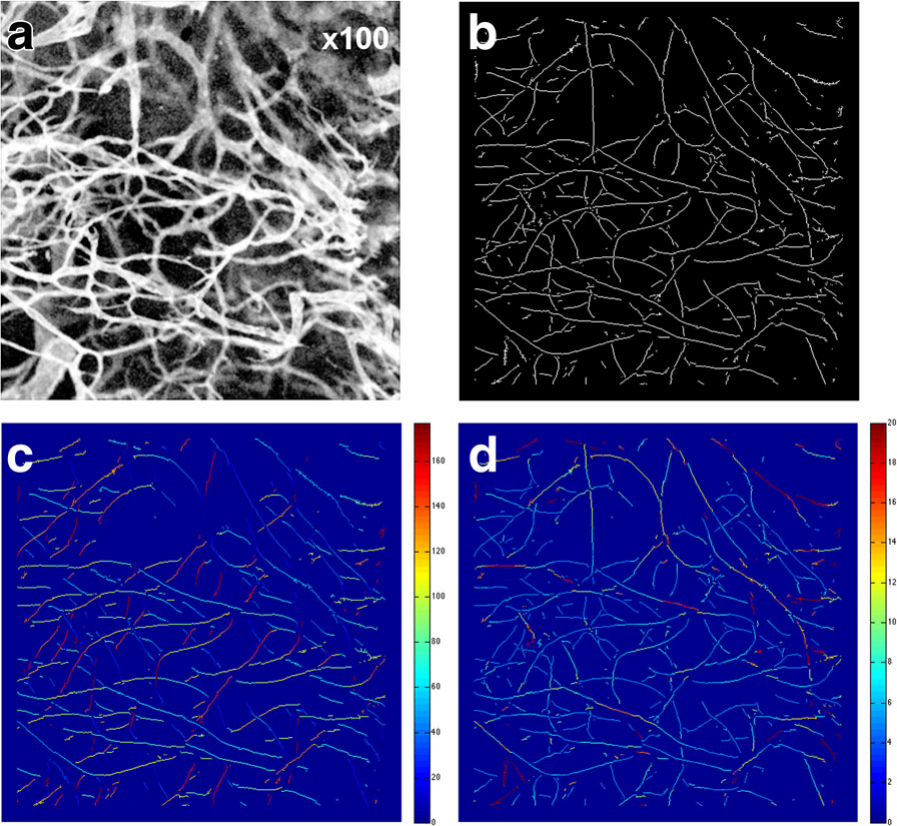
An example of vessel analysis from CD31 immunohistochemistry images. **a** Original image. **b** The binary line image. **c** Orientation image indicating the angulation of vessels (*scale* represents degrees from 0 to 180). **d** Scale image representing the size of individual vessels (*scale* represents pixels)

Phycoerythrin (PE)-conjugated anti-mouse CD31 antibody (100 μL; Biolegend) was administered intravenously 10 min before the mice were euthanised. Immediately after resection, tumour specimens were examined using confocal microscopy (Leica Microsystems Ltd) as previously described [20]. For each image (Fig. 1a), a mask of the tumour region was created and contrast of fainter vessels was improved using histogram equalisation. Vessels were detected by applying a line operator [21], non-maximal suppression [22] and binary thinning to the images. The line operator was applied with an angular resolution of 4° and over a scale range of 5–20 pixels. This resulted in three output images: a binary image showing the central line of vessels (Fig. 1b), an image showing vessel orientation (Fig. 1c) and an image showing vessel width (Fig. 1d). Vessel density was then calculated as the sum of the vessel width image divided by the area of the image mask. Mean vessel size was calculated as the sum of the vessel width image divided by the sum of the binary line image using an in-house Matlab (Mathworks) programme.

### SPECT-CT imaging

SPECT-CT imaging was performed using a nanoSPECT/CT system (Bioscan) equipped with parallel pinhole collimators. The mice (*n* = 6) were anaesthetised with isofluorane, and air mixture and imaging was conducted as reported before [23].

### Statistics

All statistical analyses were performed using Prism version 5.04 (GraphPad Software) using either two-tailed *t* tests or one-way analysis of variances (ANOVAs) as appropriate. Pearson coefficients were used to determine correlations. For the competitive binding assay, a one site-fit logIC50 curve was used. *P* values of ≤0.05 were considered statistically significant. Data are plotted as means ± standard error of the mean (SEM), based on three independent experiments.

## Results

### Radiopharmaceutical synthesis

The synthesis of radiolabelled bevacizumab was performed successfully with yields of up to 20% and very high radiochemical purity (>95%). Binding of ^111^In-bevacizumab to VEGF was unaltered compared to unmodified antibody in several (>6) competitive binding assays (Additional file 1).

### In vivo kinetics and biodistribution

^111^In-bevacizumab uptake in tumours peaked at day 5 post-injection (p.i.) (26.5 ± 3.2%ID/g, Additional file 2). In contrast, normal tissue uptake was maximal at day 1. For subsequent experiments and biodistribution imaging was performed at 5 days p.i.

**Fig. 2:**
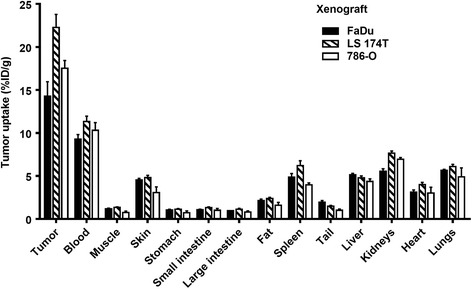
Distribution of ^111^In-bevacizumab after 5 days in mice bearing different tumour xenografts. *Black* = FaDu, *hatched* = LS 174T, *white* = 786-O. Data are presented as mean ± SEM, *n* = minimum of 4 mice/group

Uptake of ^111^In-bevacizumab at 5 days p.i. was 14.3 ± 1.7, 22.3 ± 1.5 and 17.5 ± 0.9%ID/g in FaDu, LS 174T and 786-O xenografts, respectively (Fig. 2). Apart from the blood, the uptake in all normal organs was less than 10%ID/g, yielding good visualisation of the tumour. With respect to the FaDu xenograft, there was a wide range of uptakes demonstrated with a standard deviation of 8.1, which is reflected in the variation of mean uptakes between experiments.

### Specificity

**Fig. 3:**
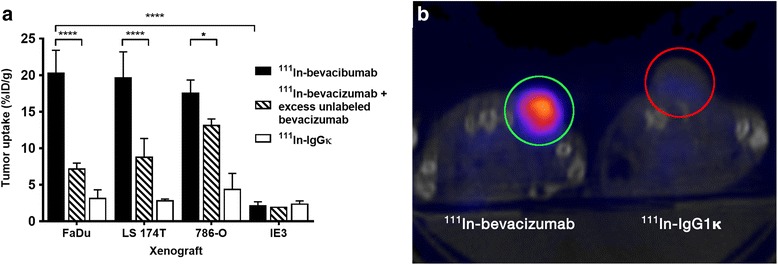
**a** Specificity of uptake of ^111^In-bevacizumab. Uptake of ^111^In-bevacizumab (*black*); ^111^In-bevacizumab + excess of unlabelled bevacizumab (*hatched*) and ^111^In-IgG1κ (*white*) in different tumour models. Data are presented as mean ± SEM, *P < 0.05; ****P < 0.0001, *n* = minimum of 4 mice/group. **b** SPECT-CT transaxial image of FaDu xenograft-bearing mice injected with either ^111^In-bevacizumab or ^111^In-IgG1κ (tumours in *circles*)

Uptake within tumours was not influenced by tumour mass (*R* = −0.06, *P* = 0.67) (Additional file 3). Tumour uptake of ^111^In-bevacizumab was greater than that of the non-specific control probe, ^111^In-IgG1κ (FaDu, 20.3 ± 1.4 versus 3.1 ± 0.5%ID/g; LS 174T, 19.6 ± 1.5 versus 2.8 ± 0.1%ID/g; 786-O, 17.5 ± 0.9 versus 4.4 ± 0.4%ID/g, *P* < 0.0001). When administered in combination with a 100-fold excess of non-radiolabelled bevacizumab, tumour uptake of ^111^In-bevacizumab was reduced compared to ^111^In-bevacizumab alone (FaDu, 20.3 ± 1.4 versus 7.2 ± 0.4%ID/g, *P* < 0.001; LS 174T, 19.6 ± 1.5 versus 8.8 ± 1.1%ID/g, *P* < 0.001; 786-O, 17.5 ± 0.9 versus 13.1 ± 0.4%ID/g, *P* < 0.05). Uptake of ^111^In-bevacizumab was low in xenografts derived from MDA-MB231 IE3 (2.1 ± 0.3%ID/g), which expresses low levels of VEGF (Fig. 3a). Taken together, these data indicate specific accumulation of ^111^In-bevacizumab in VEGF-expressing tumours. This is supported by selective SPECT-CT imaging of the mice (*n* = 6) bearing FaDu xenografts, which displayed intense uptake of tracer within the tumours in the mice injected with ^111^In-bevacizumab compared to the mice injected with ^111^In-IgG1κ (Fig. 3b).

**Fig. 4:**
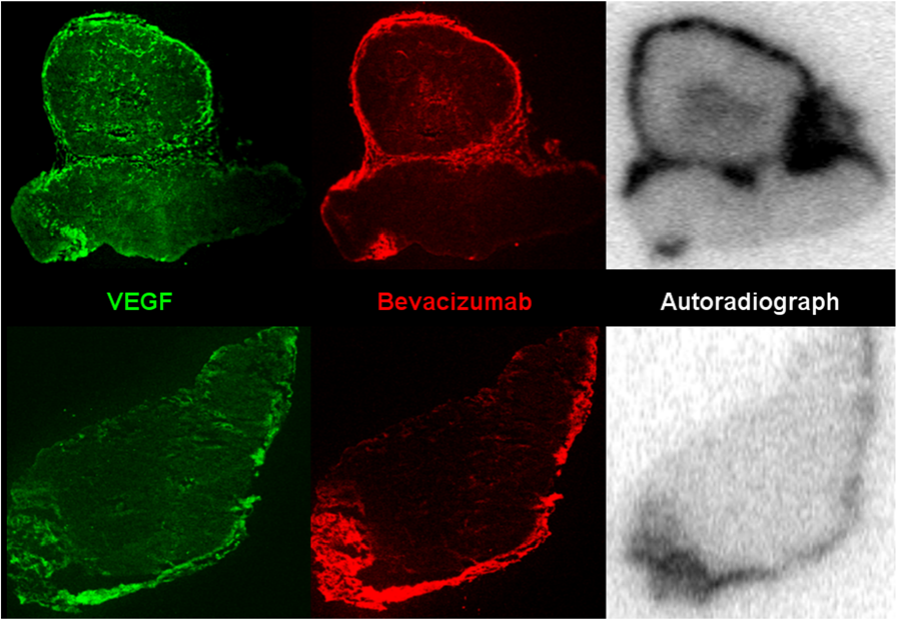
Comparison of the distribution of VEGF, bevacizumab, and ^111^In in FaDu xenograft sections. The rows are examples of two sections from different FaDu xenografts injected with ^111^In-bevacizumab and an excess of unlabelled bevacizumab at 5 days. The *left panels* (*green*) are immunofluorescence images of VEGF staining, the *centre panels* (*red*) are immunofluorescence images of bevacizumab staining, and the *right panels* (*grayscale*) are autoradiographs of ^111^In distribution

Autoradiography of tumours removed from the animals following administration of ^111^In-bevacizumab was performed to qualitatively evaluate the intratumoural distribution of ^111^In (Fig. 4). Areas of relatively high radionuclide accumulation in tumours (indicated by dark areas on the autoradiographs) showed a similar distribution to bevacizumab. This suggests that intratumoural radioactivity results from accumulation of intact radiolabelled antibody. Further evidence that the antibody has not been substantially degraded is provided by the low uptake in the spleen and liver, which would have been expected to be higher if the tracer had dissociated in vivo and released free ^111^In into the blood (Fig. 2). There was no significant correlation between uptake of ^111^In-bevacizumab and intratumoural VEGF level (Additional file 4) (*r* = 0.20, *P* = 0.19). The similar distribution of ^111^In on autoradiography with immunostaining for VEGF (Fig. 4) suggests that the tracer binds specifically to tumour-associated VEGF.

### Response to rapamycin therapy

**Fig. 5:**
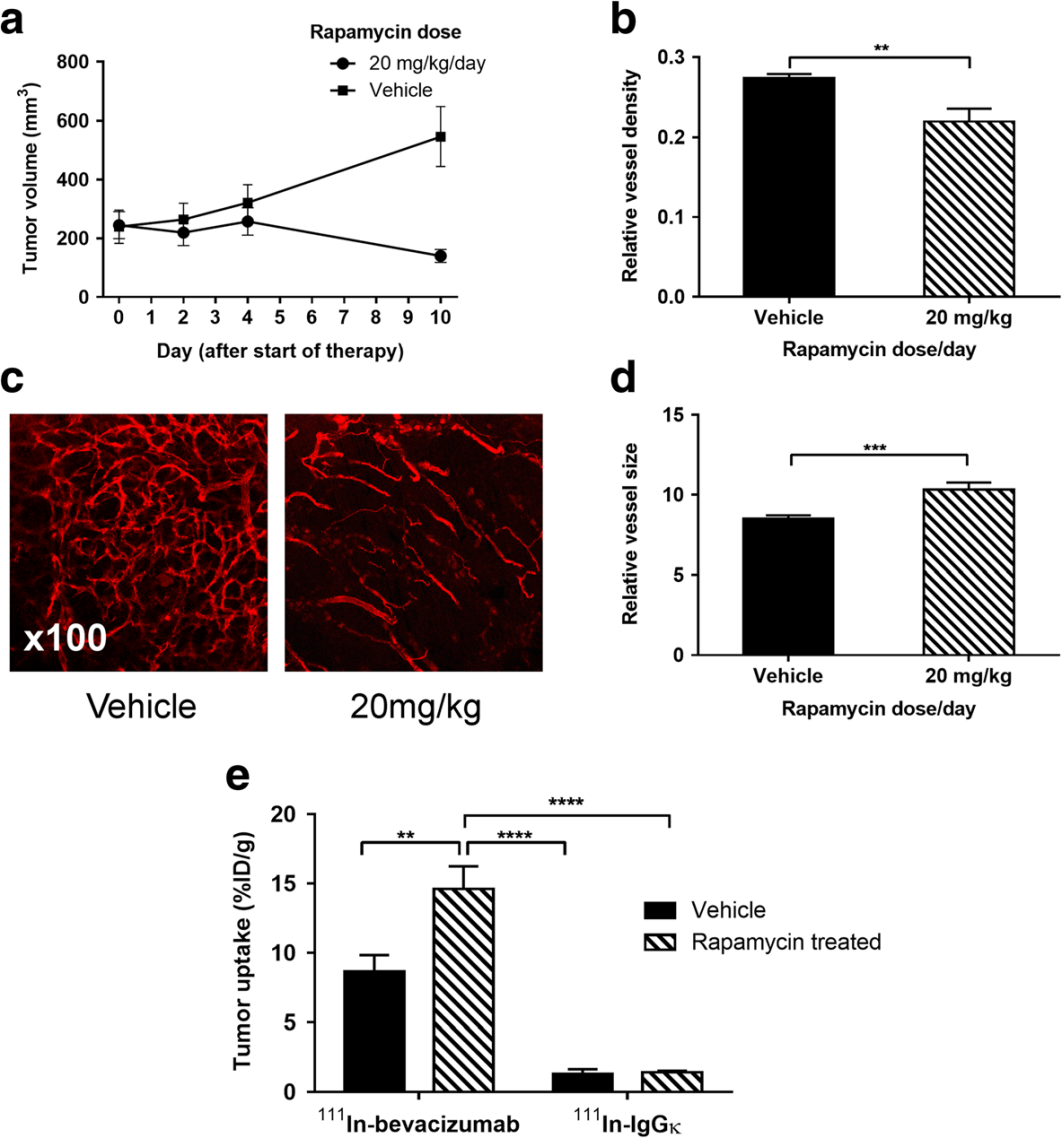
The effect of rapamycin on FaDu xenografts. **a** Changes in tumour volume with rapamycin therapy. On day 10, tumour volumes are significantly different (*P* = 0.0004). **b** Select images of CD31 confocal microscopy of tumours from mice treated with rapamycin. **c** Changes in relative mean vessel size with rapamycin therapy. **d** Changes of relative mean vessel density of rapamycin. **e** Uptake changes of ^111^In-bevacizumab and ^111^In-IgGκ in FaDu xenografts treated with rapamycin (20 mg/kg). The control groups were treated with vehicle injections. Data are presented as mean ± SEM, **P < 0.01, ***P < 0.001, ****P < 0.0001, *n* = 6–7/group

FaDu tumour xenograft-bearing mice were treated with either rapamycin (20 mg/kg daily) or vehicle for 10 days (Fig. 5). Rapamycin led to tumour growth inhibition (140 ± 22 mm^3^ versus 547 ± 101 mm^3^, *P* = 0.0004). Representative images of tumour vasculature from the two groups are shown in Fig. 5b. We found a significant decrease in relative vessel density (0.27 ± 0.01 to 0.22 ± 0.02, *P* = 0.0015) and an increase in relative mean vessel size (8.5 ± 0.2 to 10.3 ± 0.4, *P* = 0.045) compared to vehicle-treated mice. These data are consistent with vascular normalisation [24], although the extensive vessel regression observed in our model would be expected to be associated with decreased rather than increased perfusion.

Tumour uptake of ^111^In-bevacizumab significantly increased (68%, *P* < 0.01) with rapamycin therapy (Fig. 5e). In contrast, ^111^In-IgG1κ exhibited very low uptake, which did not significantly change (*P* > 0.05) after rapamycin therapy, indicating that the increase in ^111^In-bevacizumab uptake is specific and not related to vascular changes.

**Fig. 6:**
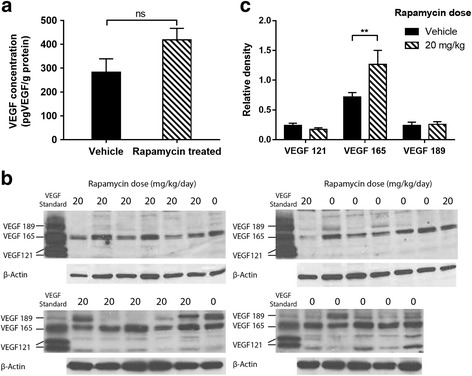
The effect of rapamycin therapy on VEGF levels in FaDu xenografts. **a** Changes in VEGF levels as measured by ELISA. Differences in levels were not statistically significant. **b** Changes in VEGF isoforms assessed by Western blot in FaDu xenografts treated with either rapamycin (20 mg/kg/day) or vehicle control for 10 days. **c** Changes in VEGF isoforms were measured using densitometry in the corresponding blots. Data are presented as mean ± SEM, **P < 0.01, *n* = 11/group

Comparison with overall VEGF levels (measured by ELISA) within the tumours suggested that the increased uptake of ^111^In-bevacizumab was associated with an increase in VEGF (Fig. 6a) (from 283 ± 56 to 419 ± 47 pg/g of protein) measured by ELISA; however, this did not reach statistical significance (*P* = 0.07). This may be explained by analysis of Western blots (Fig. 6b), which demonstrated a significant increase in VEGF_165_ (*P* < 0.01) but no significant change in the VEGF_121_ and VEGF_189_ isoforms (Fig. 6c).

## Discussion

Great progress has been made in the development of anti-angiogenic therapies targeting VEGF or its upstream targets, such as phosphoinositide 3-kinase (PI3K), mTOR and AKT [19, 24, 25]. However, these agents are only effective in a small and, at present, unpredictable subset of patients. This fact, coupled with high drug costs and adverse side effects has highlighted the need for accurate biomarkers for assessing angiogenesis and its response to therapy. Hence, non-invasive monitoring of angiogenesis by imaging is urgently needed to better assess and modify anti-angiogenic therapies. Successful implementation of imaging and treatment monitoring using radiotracers requires detailed knowledge of their pharmacokinetics and specificity. In this context, we investigated the role of ^111^In-bevacizumab in detecting response to anti-angiogenic therapy targeting mTOR.

Preclinical studies have provided evidence for the specific binding of ^111^In-bevacizumab to VEGF [16, 26]. However, in clinical trials involving patients with renal cell carcinoma (14 patients) [18] and metastatic colorectal cancer (12 patients) [27], no correlation was observed between uptake of tracer and intratumoural VEGF expression level, measured using either enzyme-linked immunosorbent assays (ELISAs), in situ hybridisation (ISH) or immunohistochemistry (IHC). In contrast, in a study of ^111^In-bevacizumab used in patients with melanoma [17], correlation between uptake of the tracer in the tumours and IHC of VEGF in tumour specimens was observed (nine patients).

Pharmacokinetic analyses in our study demonstrated that the greatest uptake and greatest tumour to background ratio occurs at around 5 days after injection. This differs from the data reported by Stollman et al. [16] who showed in the same murine tumour model that uptake was greatest on day 3 and the tumour to background ratios increased over the week with day 7 post-injection having the highest values. However, their work did not measure uptake values on day 5 and so may have missed the peak signal to background ratio. The overall prediction of our pharmacokinetics is that in patients, the optimal time for imaging would be 3–5 days post-injection. Our study corroborates a recent clinical study that demonstrated best visualisation and quantification of ^111^In-bevacizumab is 4 days post-injection in melanoma patients [17].

We showed that ^111^In-bevacizumab accumulated specifically in tumours. This was demonstrated in three VEGF-expressing xenograft models by suppression of uptake through competitive inhibition by an excess of unlabelled bevacizumab and by comparison with an ^111^In-labelled IgG isotype-matched control antibody. A direct relationship to VEGF, however, was not supported by comparing uptake with VEGF ELISAs of the tumour tissue. VEGF has many isoforms, and there are differences in their concentration, localisation and affinity for bevacizumab. Thus, a single ELISA or indeed other methods may not correlate with localisation of the antibody. Indeed, it may be that antibody uptake is the most important clinical variable to study for response, rather than VEGF and all its isoforms. We hypothesised that different isoforms of VEGF detected by ^111^In-bevacizumab would be predominantly matrix and cell membrane-associated VEGF (e.g., VEGF_165_ and VEGF_189_), whereas ELISA measures all isoforms, including diffusible forms such as VEGF_121_ [26]. This is supported by the spatial distribution of VEGF observed in tumour histological sections that co-localised with the tracer on autoradiography. In addition, uptake of ^111^In-bevacizumab has been shown to be only specific in blocking studies with tumours expressing VEGF_165_ and VEGF_189_ in comparison to those expressing VEGF_121_ [26]. Our results also are in agreement with the reports of recent clinical trials with^111^In-bevacizumab in patients with colorectal cancer liver metastases and renal cell carcinoma (RCC) that failed to identify a correlation between uptake of ^111^In-bevacizumab and VEGF levels measured by ELISA and ISH [18, 27]. Altogether, these data build on previous evidence supporting that circulating VEGF measurements have been of little value in monitoring or predicting response to bevacizumab in previous studies [28].

For the assessment of response of ^111^In-bevacizumab uptake to anti-angiogenic therapy, we used the mTOR inhibitor rapamycin at a dose previously demonstrated to have strong anti-angiogenic activity [29, 30]. Rapamycin led to significant tumour growth delay associated with profound vascular regression as reported before [31]. Therefore, the increased uptake of ^111^In-bevacizumab after treatment with rapamycin therapy is unlikely to be due to improved perfusion due to the strong anti-angiogenic effect. This contrasts with a prior clinical study where treatment with sorafenib, a multikinase inhibitor targeting mainly VEGF receptors, in RCC led to decreased ^111^In-bevacizumab uptake and did not correlate with VEGF levels [18]. This was hypothesised to be due to destruction of the vasculature, preventing tracer delivery to the tumour. This discrepancy is difficult to interpret, though may relate to the relative small size of tumours in mouse models compared to patients, where diffusion of the radiotracer may potentially overcome poor delivery by destroyed vasculature.

In our work, quantification of VEGF levels demonstrated a trend towards increased VEGF levels post-rapamycin, associated with an increase in uptake of ^111^In-bevacizumab. The lack of statistical significance in the increase of overall VEGF levels could be attributed to relative differences in VEGF isoforms. Indeed, the changes in VEGF levels were predominantly due to an increase in VEGF_165_, which may reflect the importance of VEGF_165_ over other isoforms in tumour angiogenesis [32]. VEGF_121_ and VEGF_189_ levels did not appear to change with rapamycin therapy. These results are surprising as they conflict with the mechanistic hypothesis and experimental evidence that mTOR inhibition decreases VEGF levels [30, 33, 34]. In particular, a similar study by van der Bilt et al. [35] used ^89^Zr-bevacizumab to assess response to everolimus in ovarian cancer xenografts. They showed that VEGF-A and ^89^Zr-bevacizumab uptake decreased in the xenografts after treatment with 10 mg/kg of everolimus. A potential explanation for this contrast is the dose of rapamycin used in our study could have led to increase in hypoxia by vascular regression and hence VEGF induction via activation of HIF-1α. Alternatively, it is well recognised that inhibition of mammalian target of rapamycin complex 1 (mTORC1) by rapamycin can result in a feedback upregulation of AKT and PI3K pathways [36, 37], and both pathways are known to increase VEGF expression [19, 38]. So it will be interesting in future studies to use ^111^In-bevacizumab to compare drugs that block both mTOR and this feedback activation.

## Conclusions

Collectively, these data show that ^111^In-bevacizumab is a specific radiotracer to visualise VEGF within tumours and monitor response to anti-angiogenic therapy mediated by mTOR inhibition. ^111^In-bevacizumab was specific for matrix and cell membrane-associated forms of VEGF. This work provides important insight and support for further exploring ^111^In-bevacizumab for non-invasive imaging of VEGF in the clinical setting.

## Additional files

Additional file 1: Competitive binding of ^111^In-bevacizumab to VEGF. The IC50 value for unlabeled bevacizumab was 0.84 ± 0.45 nM when competed with 1 nM of ^111^In-bevacizumab for binding to cells expressing VEGF. Data are presented as mean ± SEM, *n* = 12.

Additional file 2: Kinetics of uptake of ^111^In-bevacizumab in LS174T xenografts and organs in female BALB/c nude mice. ANOVA demonstrate a statistically significant difference between the means of each day (*P* = 0.03). Error bars are SEM, *n* = 4/group.

Additional file 3: Comparison of tumour mass with uptake of ^111^In-bevacizumab. There is no correlation.

Additional file 4: Comparison of VEGF in tumours, as measured by ELISA, with uptake of ^111^In-bevacizumab. There is no correlation.

## Abbreviations

AKT: Protein kinase B
ANOVA: Analysis of variance
Bis-Tris: [Bis (2-hydroxyethyl) imino-tris (hydroxymethyl) methane-HCl]
DMEM: Dulbecco’s modified Eagle’s medium
DMSO: Dimethyl sulfoxide
DTPA: Diethylenetriamine pentaacetic acid
ECM: Extracellular matrix
EDTA: Ethylenediaminetetraacetic acid
ELISA: Enzyme-linked immunosorbent assay
FBS: Foetal bovine serum
HEPES: 4-(2-Hydroxyethyl)-1-piperazineethanesulfonic acid
HRP: Horseradish peroxidase
IC_50_: Half maximal inhibitory concentration
IgG: Immunoglobulin G
IHC: Immunohistochemistry
ISH: In situ hybridisation
ITLC: Instant thin layer chromatography
mTOR: Mammalian target of rapamycin
mTORC1: Mammalian target of rapamycin complex 1
PEG: Polyethylene glycol
PI3K: Phosphoinositide 3-kinase
RCC: Renal cell cancer
SEM: Standard error of the mean
VEGF: Vascular endothelial growth factor
VEGFR: VEGF receptor
