# Supplementary figures and images for: Monitoring response to anti-angiogenic mTOR inhibitor therapy in vivo using 111In-bevacizumab
Source: EJNMMI Res. 2017 May 30;7:49. doi: 10.1186/s13550-017-0297-9 (PMC5449352; doi:10.1186/s13550-017-0297-9)

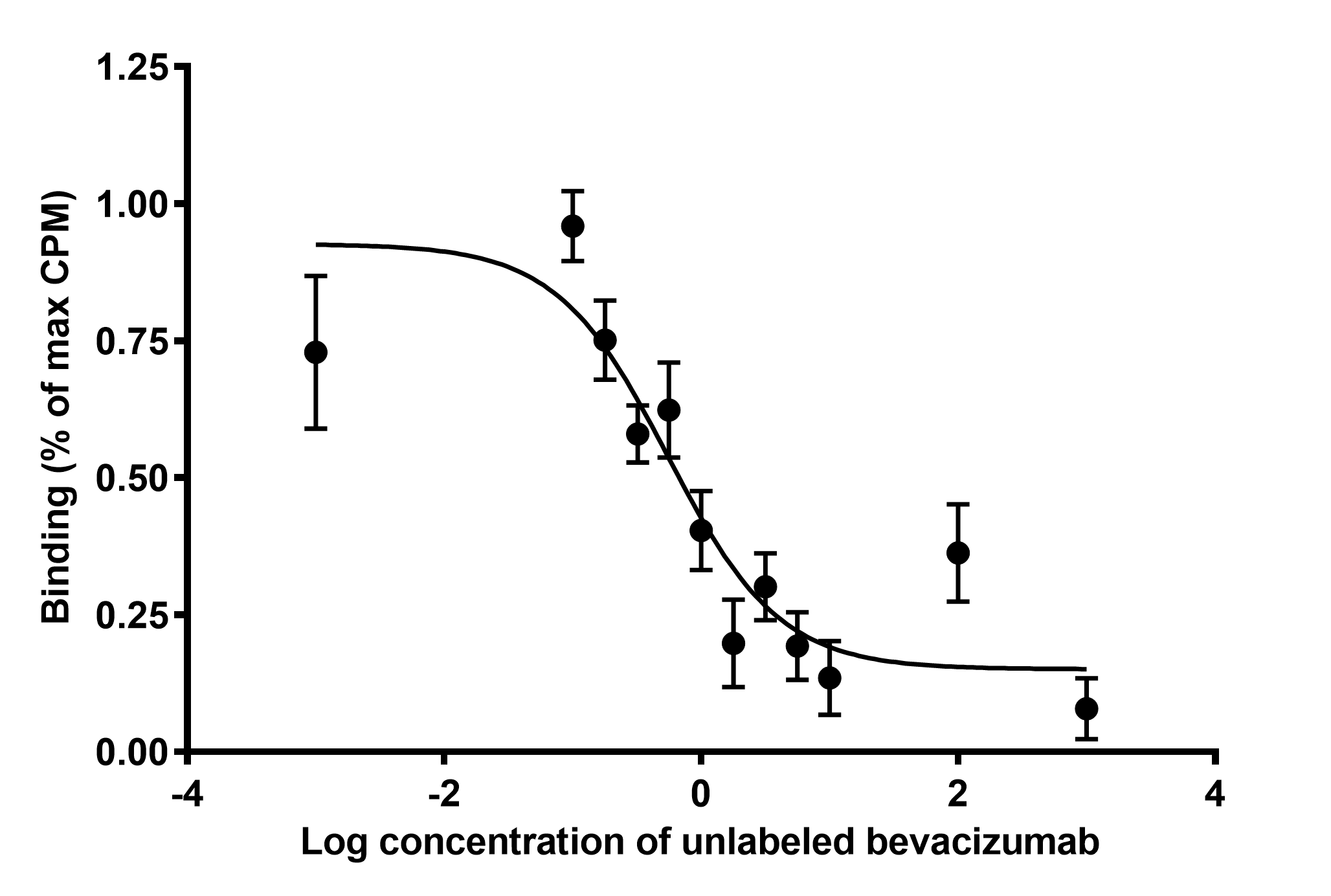

Supplement: Supplementary file 1 — Competitive binding of 111In-bevacizumab to VEGF. The IC50 value for unlabeled bevacizumab was 0.84 ± 0.45 nM when competed with 1 nM of 111In-bevacizumab for binding to cells expressing VEGF. Data are presented as mean ± SEM, n = 12. [file 13550_2017_297_MOESM1_ESM.tif]

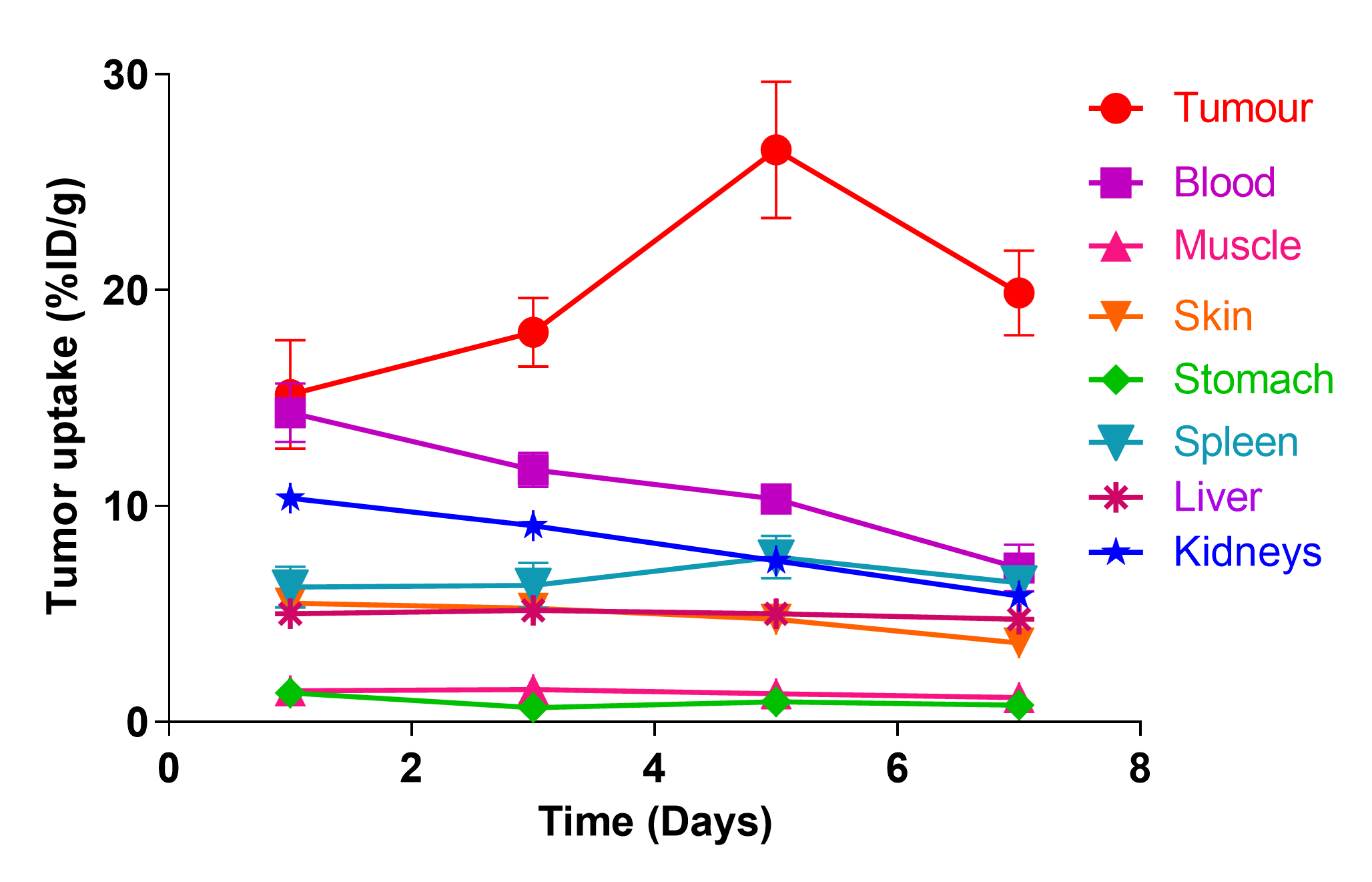

Supplement: Supplementary file 2 — Kinetics of uptake of 111In-bevacizumab in LS174T xenografts and organs in female BALB/c nude mice. ANOVA demonstrate a statistically significant difference between the means of each day (P = 0.03). Error bars are SEM, n = 4/group. [file 13550_2017_297_MOESM2_ESM.tif]

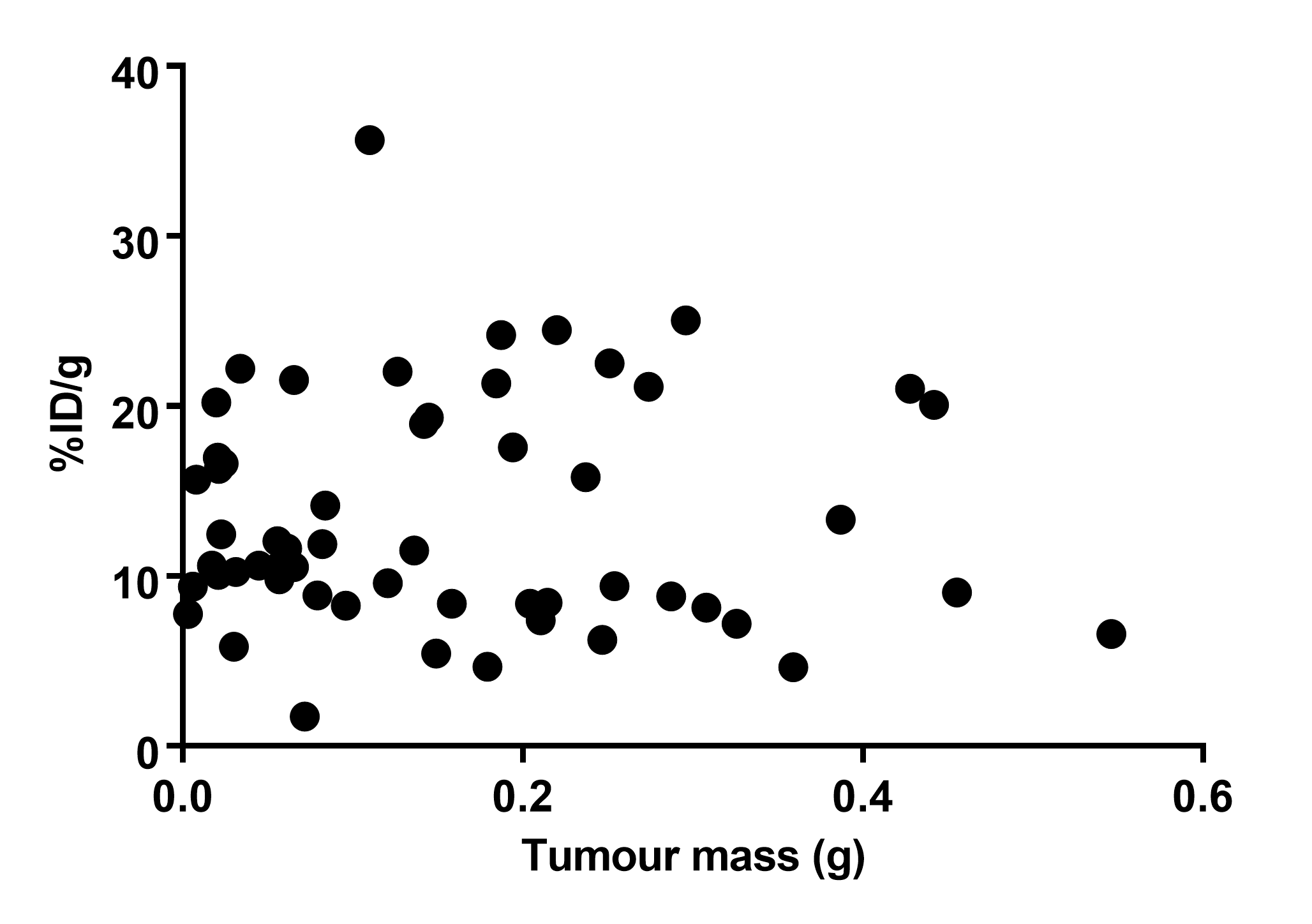

Supplement: Supplementary file 3 — Comparison of tumour mass with uptake of 111In-bevacizumab. There is no correlation. [file 13550_2017_297_MOESM3_ESM.tif]

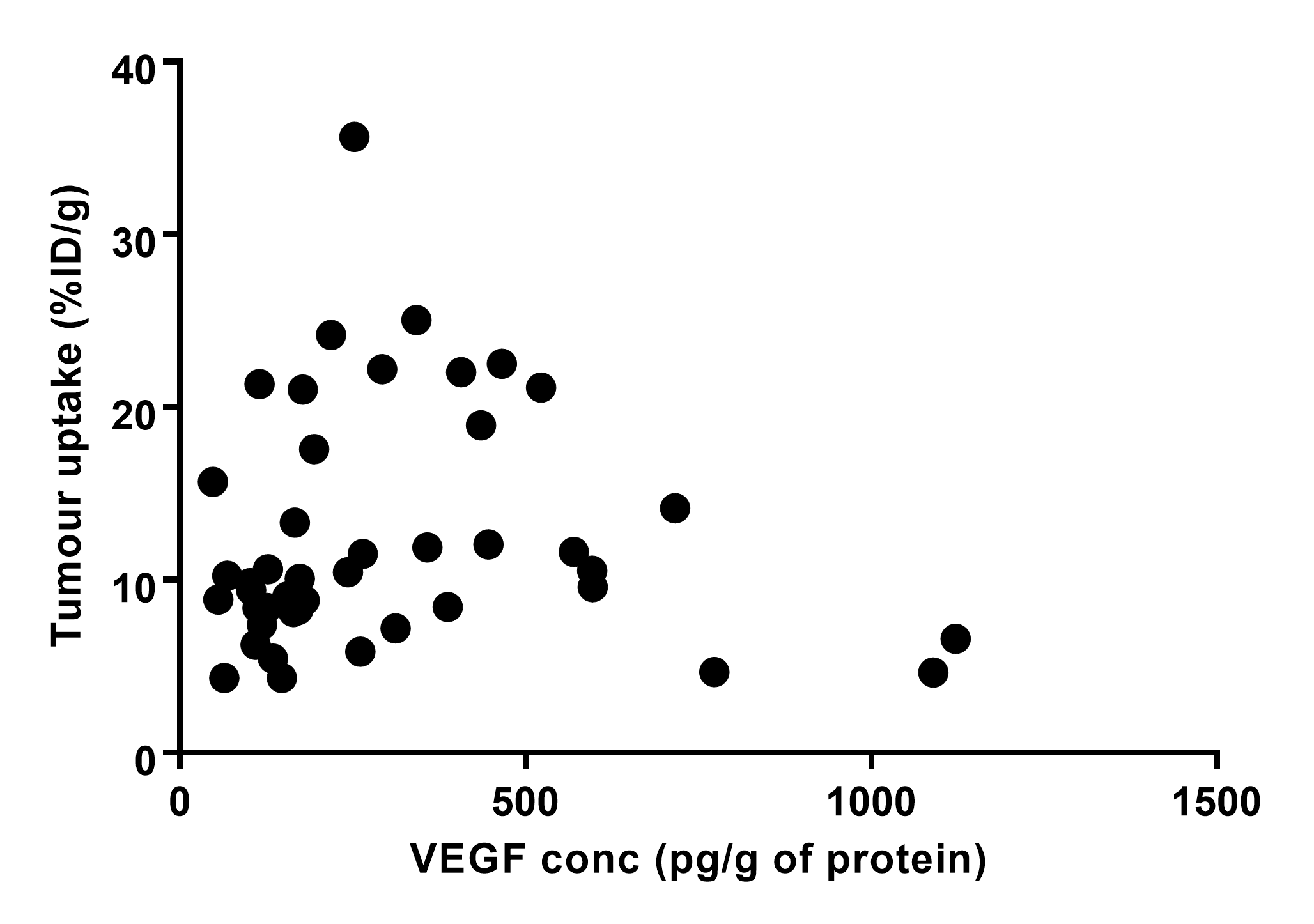

Supplement: Supplementary file 4 — Comparison of VEGF in tumours, as measured by ELISA, with uptake of 111In-bevacizumab. There is no correlation. [file 13550_2017_297_MOESM4_ESM.tif]
